# Supplementary material for: Monitoring the Dilution of Buffer Solutions with Different pH Values above and below Physiological pH in Very Small Volumes
Source: Sensors (Basel). 2024 Sep 4;24(17):5751. doi: 10.3390/s24175751 (PMC11487416; doi:10.3390/s24175751)
Supplement: Supplementary file 1 [file sensors-24-05751-s001.zip › sensors-3137961-supplementary.pdf]

sensors

MDPI

Supplementary Materials for

Monitoring the Dilution of Buffer Solutions with Different pH Values above and below Physiological pH in Very Small Volumes

Vinayak J. Bhat [1,2,*], Daniel Blaschke [1,*], Sahitya V. Vegesna [1,2], Sindy Burgold-Voigt [1,3], Elke Müller [1,3], Ralf Ehricht [1,3,4] and Heidemarie Schmidt [1,2,*]

[1] Leibniz Institute of Photonic Technology, 07745 Jena, Germany; sahityav.vegesna@leibniz-ipht.de (S.V.V.)
[2] Institute of Solid State Physics, Friedrich Schiller University Jena, 07743 Jena, Germany
[3] InfectoGnostics Research Campus, 07743 Jena, Germany
[4] Institute of Physical Chemistry, Friedrich Schiller University Jena, 07743 Jena, Germany
* Correspondence: vinayak.bhat@uni-jena.de (V.J.B.); daniel.blaschke@leibniz-ipht.de (D.B.); heidemarie.schmidt@uni-jena.de (H.S.)

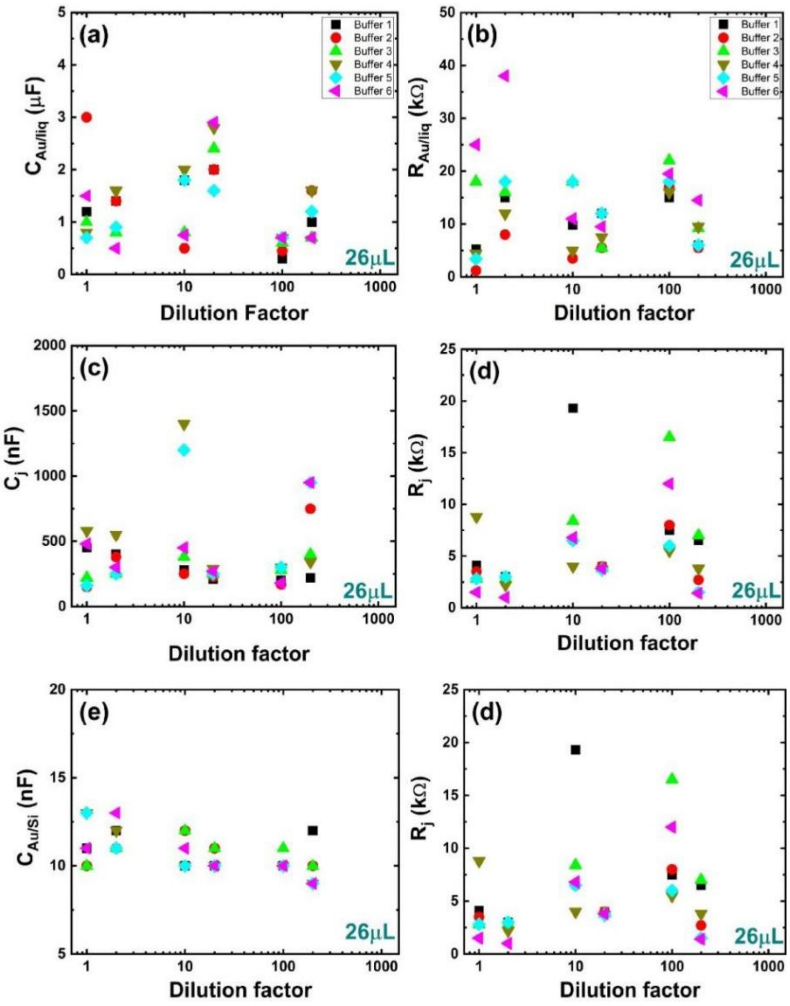

Figure S1. Modelling values of parameters related to the buffer-silicon surface-gold ring electrode in the equivalent circuit model: (a) $C_{Au/liq}$, (b) $R_{Au/liq}$, (c) C_j, (d) R_j, (e) $C_{Au/Si}$, and (f) $R_{liq,}$ plotted against the dilution factor of buffers. Buffer test solution 1 is represented by black square dots, buffer 2 by red circle dots, buffer 3 by green triangle dots, buffer 4 by dark green down triangle dots, buffer 5 by cyan diamond dots, and buffer 6 by magenta side triangle dots. The values are plotted for 26μL volume added inside the ring electrode. These parameters shows variations with the change in pH, volume and dilution concentration.

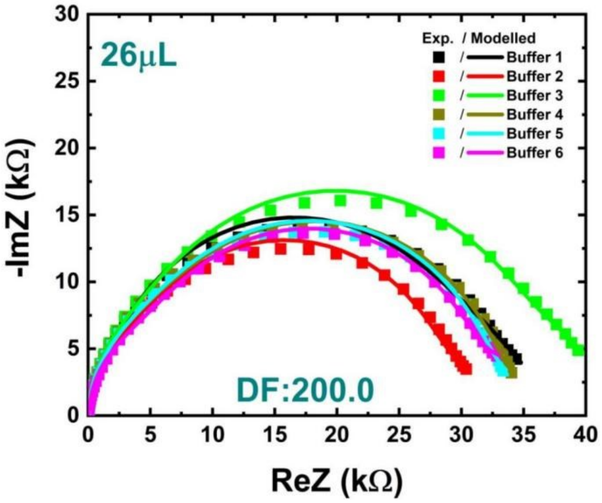

Figure S2. Measured and modeled Nyquist plot of a boron-doped silicon impedance chip with 26 μL of buffer added inside the top ring electrode. The plot represents the diluted buffers with a dilution factor of 200. The measured data points are indicated by dots, while the modeled results are represented by solid lines.

Table S1. Equivalent circuit parameter values for the empty impedance and obtained modelling parameters for the buffer test solution 1 (PBS s.f.) with the initial concentration (dilution factor 1.0) and later diluted to dilution factor of 2.0, 10.0, 20.0, 100.0, and 200.0. These values are obtained for added 26 μL of buffer volume inside the ring electrode. The cut-off frequency of the buffer-silicon-ring electrode-related parameters is below 40 Hz during empty impedance to ensure no effect of these parameters during empty modelling.

Buffer 1	Top contact							p^+n junction			Bottom contact	
	$C_{Au/liq}$ (μF)	$R_{Au/liq}$ (kΩ)	Cj (nF)	Rj (kΩ)	R_{liq} (kΩ)	$C_{Au/Si(p+)}$ (nF)	$R_{Au/Si(p+)}$ (kΩ)	C_{p+n} (nF)	R_{p+n} (kΩ)	Rs (Ω)	$C_{Si(n)/Au}$ (nF)	$R_{Si(n)/Au}$ (Ω)
						26 μL						
Empty	20.0	0.18	100	2.8	1.6	17.0	11.0	6.1	28	8	17	20.0
1.0	1.2	5.2	450	4.1	2.6	11.0	22.0	9.8	29.3	7	15	40.0
2.0	1.4	15.0	400	3.0	0.3	12.0	15.0	10.2	31.2	9	9	50.0
10.0	1.8	9.8	280	9.3	2.7	10.0	16.0	10.5	33.8	6	15	30.0
20.0	2.0	12.0	210	4.0	1.1	10.0	25.0	10.4	30	9	14	34.0
100.0	0.3	15.0	200	7.5	4.0	10.0	21.0	8.2	29.5	6	16	35.0
200.0	1.0	6.0	220	6.5	2.8	12.0	26.0	7.5	27.2	16	12	20.0

Table S2. Equivalent circuit parameter values for the empty impedance and obtained modelling parameters for the buffer test solution 2 (LB) with the initial concentration (dilution factor 1.0) and later diluted to dilution factor of 2.0, 10.0, 20.0, 100.0, and 200.0. These values are obtained for added 26 μL of buffer volume inside the ring electrode. The cut-off frequency of the buffer-silicon-ring electrode-related parameters is below 40 Hz during empty impedance to ensure no effect of these parameters during empty modelling.

Buffer 2	Top contact							p^+n junction			Bottom contact	
	$C_{Au/liq}$ (μF)	$R_{Au/liq}$ (kΩ)	Cj (nF)	Rj (kΩ)	R_{liq} (kΩ)	$C_{Au/Si(p+)}$ (nF)	$R_{Au/Si(p+)}$ (kΩ)	C_{p+n} (nF)	R_{p+n} (kΩ)	Rs (Ω)	$C_{Si(n)/Au}$ (nF)	$R_{Si(n)/Au}$ (Ω)
						26 μL						
Empty	20.0	0.18	100	2.8	1.6	17.0	11.0	6.1	28.0	8.0	17.0	20.0
1.0	3.0	1.2	150	3.5	1.8	10.0	11.0	7.0	29.0	15.0	17.0	30.0
2.0	1.4	8.0	380	2.6	0.8	11.0	15.0	10.2	33.9	9.0	9.0	40.0
10.0	0.5	3.5	250	6.5	1.2	12.0	18.0	9.0	34.0	8.0	14.0	42.0
20.0	2.0	5.5	230	4.0	1.5	11.0	23.0	10.5	31.1	9.0	13.0	50.0
100.0	0.4	17.0	170	8.0	4.8	10.0	22.0	9.0	28.3	8.0	16.0	28.0
200.0	1.6	5.5	750	2.7	5.0	10.0	23.0	9.8	24.2	9.0	15.0	45.0

Table S3. Equivalent circuit parameter values for the empty impedance and obtained modelling parameters for the buffer test solution 3 (LB filtered / 0.2 μm CA filter) with the initial concentration (dilution factor 1.0) and later diluted to dilution factor of 2.0, 10.0, 20.0, 100.0, and 200.0. These values are obtained for added 26 μL of buffer volume inside the ring electrode. The cut-off frequency of the buffer-silicon-ring electrode-related parameters is below 40 Hz during empty impedance to ensure no effect of these parameters during empty modelling.

	**Top contact**							**p^+n junction**			**Bottom contact**	
**Buffer 3**	**$C_{Au/liq}$ (μF)**	**$R_{Au/liq}$ (kΩ)**	**Cj (nF)**	**Rj (kΩ)**	**R_{liq} (kΩ)**	**$C_{Au/Si(p+)}$ (nF)**	**$R_{Au/Si(p+)}$ (kΩ)**	**C_{p+n} (nF)**	**R_{p+n} (kΩ)**	**Rs (Ω)**	**$C_{Si(n)/Au}$ (nF)**	**$R_{Si(n)/Au}$ (Ω)**
						**26 μL**						
Empty	20.0	0.18	100	2.8	1.6	17.0	11.0	6.1	28.0	8.0	17.0	20.0
1.0	1.0	18.0	220	2.8	0.9	10.0	13.0	10.0	27.3	9.0	10.0	50.0
2.0	0.8	16.0	250	2.8	0.8	11.0	12.0	10.2	31.4	9.0	9.0	85.0
10.0	0.8	18.0	380	8.4	2.5	12.0	17.0	8.8	34.8	8.0	14.0	48.0
20.0	2.4	5.5	250	3.8	1.8	11.0	18.0	10.6	30.0	9.0	15.0	60.0
100.0	0.60	22.0	280	16.5	4.8	11.0	14.0	9.5	28.5	8.0	13.0	50.0
200.0	0.7	9.2	400	7.0	6.0	10.0	22.0	8.6	30.9	9.0	15.0	40.0

Table S4. Equivalent circuit parameter values for the empty impedance and obtained modelling parameters for the buffer test solution 4 (0.1M $NaHCO_3$ + NAP-5) with the initial concentration (dilution factor 1.0) and later diluted to dilution factor of 2.0, 10.0, 20.0, 100.0, and 200.0. These values are obtained for added 26 μL of buffer volume inside the ring electrode. The cut-off frequency of the buffer-silicon-ring electrode-related parameters is below 40 Hz during empty impedance to ensure no effect of these parameters during empty modelling.

	**Top contact**							**p^+n junction**			**Bottom contact**	
**Buffer 4**	**$C_{Au/liq}$ (μF)**	**$R_{Au/liq}$ (kΩ)**	**Cj (nF)**	**Rj (kΩ)**	**R_{liq} (kΩ)**	**$C_{Au/Si(p+)}$ (nF)**	**$R_{Au/Si(p+)}$ (kΩ)**	**C_{p+n} (nF)**	**R_{p+n} (kΩ)**	**Rs (Ω)**	**$C_{Si(n)/Au}$ (nF)**	**$R_{Si(n)/Au}$ (Ω)**
						**26 μL**						
Empty	20.0	0.18	100	2.8	1.6	17.0	11.0	6.1	28.0	8.0	17.0	20.0
1.0	0.8	4.4	580	8.8	0.9	13.0	11.0	10.5	29.2	9.0	13.0	55.0
2.0	1.6	12.0	550	2.2	1.0	12.0	12.0	10.8	32.2	9.0	9.0	95.0
10.0	2.0	5.00	1400	4.0	2.5	10.0	18.0	10.8	28.8	9.0	13.0	80.0
20.0	0.30	15.00	200	7.5	4.0	10.0	21.0	8.2	29.5	6.0	16.0	35.0
100.0	0.7	16.0	300	5.5	4.5	10.0	20.0	9.5	29.0	8.0	16.0	40.0
200.0	1.6	9.5	340	3.8	8.0	9.0	22.0	10.0	26.1	9.0	12.0	35.0

Table S5. Equivalent circuit parameter values for the empty impedance and obtained modelling parameters for the buffer test solution 5 (0.1M $NaHCO_3$) with the initial concentration (dilution factor 1.0) and later diluted to dilution factor of 2.0, 10.0, 20.0, 100.0, and 200.0. These values are obtained for added 26 μL of buffer volume inside the ring electrode. The cut-off frequency of the buffer-silicon-ring electrode-related parameters is below 40 Hz during empty impedance to ensure no effect of these parameters during empty modelling.

	**Top contact**							**p^+n junction**			**Bottom contact**	
**Buffer 5**	**$C_{Au/liq}$ (μF)**	**$R_{Au/liq}$ (kΩ)**	**Cj (nF)**	**Rj (kΩ)**	**R_{liq} (kΩ)**	**$C_{Au/Si(p+)}$ (nF)**	**$R_{Au/Si(p+)}$ (kΩ)**	**C_{p+n} (nF)**	**R_{p+n} (kΩ)**	**Rs (Ω)**	**$C_{Si(n)/Au}$ (nF)**	**$R_{Si(n)/Au}$ (Ω)**
						**26 μL**						
Empty	20.0	0.18	100	2.8	1.6	17.0	11.0	6.1	28.0	8.0	17.0	20.0
1.0	0.7	3.4	160	2.8	2.2	13.0	11.0	9.0	27.3	9.0	13.0	70.0
2.0	0.9	18.0	250	3.0	1.2	11.0	12.0	10.8	34.1	9.0	9.0	90.0
10.0	1.8	18.0	1200	6.5	1.5	10.0	16.0	10.2	32.5	8.0	12.0	80.0
20.0	1.6	12.0	250	3.7	2.1	10.0	25.0	10.7	34.0	9.0	13.0	70.0
100.0	0.7	18.0	300	6.0	6.0	10.0	24.0	8.6	30.4	8.0	16.0	45.0
200.0	1.2	6.0	950	1.5	6.5	9.0	25.0	9.6	28.8	19.0	10.0	35.0

Table S6. Equivalent circuit parameter values for the empty impedance and obtained modelling parameters for the buffer test solution 6 (LB filtered / 0.2 μm CA filter + NAP-5 + 0.1M $NaHCO_3$) with the initial concentration (dilution factor 1.0) and later diluted to dilution factor of 2.0, 10.0, 20.0, 100.0, and 200.0. These values are obtained for added 26 μL of buffer volume inside the ring electrode. The cut-off frequency of the buffer-silicon-ring electrode-related parameters is below 40 Hz during empty impedance to ensure no effect of these parameters during empty modelling.

**Buffer 6**	**Top contact**							**p⁺n junction**			**Bottom contact**	
	**$C_{Au/liq}$ (μF)**	**$R_{Au/liq}$ (kΩ)**	**Cj (nF)**	**Rj (kΩ)**	**R_{liq} (kΩ)**	**$C_{Au/Si(p+)}$ (nF)**	**$R_{Au/Si(p+)}$ (kΩ)**	**C_{p+n} (nF)**	**R_{p+n} (kΩ)**	**Rs (Ω)**	**$C_{Si(n)/Au}$ (nF)**	**$R_{Si(n)/Au}$ (Ω)**
						**26 μL**						
Empty	20.0	0.18	100	2.8	1.6	17.0	11.0	6.1	28.0	8.0	17.0	20.0
1.0	1.5	25.0	480	1.5	0.9	11.0	12.0	11.0	30.8	18.0	8.0	30.0
2.0	0.5	38.0	300	1.0	1.0	13.0	10.0	11.3	30.1	9.0	8.0	90.0
10.0	0.8	11.0	450	6.8	2.0	11.0	16.0	9.3	28.3	8.0	17.0	40.0
20.0	2.9	9.5	270	3.8	2.7	10.0	21.0	10.8	31.1	9.0	16.0	60.0
100.0	0.7	19.5	180	12.0	5.5	10.0	22.0	9.1	27.7	6.0	16.0	35.0
200.0	0.7	14.5	950	1.4	6.8	9.0	23.0	11.7	25.7	9.0	18.0	75.0
